# Supplementary material for: Effect of Embryo Vitrification on the Steroid Biosynthesis of Liver Tissue in Rabbit Offspring
Source: Int J Mol Sci. 2020 Nov 16;21(22):8642. doi: 10.3390/ijms21228642 (PMC7696440; doi:10.3390/ijms21228642)
Supplement: Supplementary file 1 [file ijms-21-08642-s001.zip › ijms-961516-supplementary.docx]

**Supplementary Table 1. Details of primers used for qPCR.**

| **Gen symbol** | **Accession number** | **Forward primer** | **Reverse primer** | **Fragment (bp)** |
| --- | --- | --- | --- | --- |
| APOA4 | ENSOCUG00000011233 | ACTTCTCGCAGCTGAGCAAC | AAAGTACCACCATAAAGCAGCA | 98 |
| LIPC | ENSOCUT00000001646 | ATGCAGAAAATCCCCATCAC | CCAAATTCAGCGTGATCAGA | 79 |
| CLN6 | ENSOCUT00000017532 | AGAACTGGGTGCTGGACTTC | ATGACGTTGTAGGCCATGTG | 115 |
| ELOVL4 | ENSOCUG00000005334 | CATGGGATCATATAATGCAGGA | AAAGTACCACCATAAAGCAGCA | 103 |
| CYP7A1 | ENSOCUT00000012281 | GCCCTGAAAGCAGCTACTGA | GGGACTCCTTGATGATGCTG | 151 |
| IGF-I | NM_001082026.1 | TGGTGGATGCTCTTCAGTTCGTGT | GCTGATACTTCTGAGTCTTGGGCA | 237 |
| H2AFZ | AF030235 | AGAGCCGGCTGCCAGTTCC | CAGTCGCGCCCACACGTCC | 85 |
| GAPDH | L23961 | GCCGCTTCTTCTCGTGCAG | ATGGATCATTGATGGCGACAACAT | 144 |

APOA4: Apolipoprotein A-IV. ELOVL4: Fatty Acid Elongase 4. LIPC: Lipase C. CLN6: Transmembrane ER Protein. IGF-I: Insulin-like growth factor I. H2AFZ, H2A histone family member Z. GAPDH: Glyceraldehyde-3-phosphate dehydrogenase.
